# Nascent chain dynamics and ribosome interactions within folded ribosome–nascent chain complexes observed by NMR spectroscopy†

**DOI:** 10.1039/d1sc04313g

**Published:** 2021-09-09

**Authors:** Charles Burridge, Christopher A. Waudby, Tomasz Włodarski, Anaïs M. E. Cassaignau, Lisa D. Cabrita, John Christodoulou

**Affiliations:** Institute of Structural and Molecular Biology, University College London London WC1E 6BT UK c.waudby@ucl.ac.uk j.christodoulou@ucl.ac.uk

## Abstract

The folding of many proteins can begin during biosynthesis on the ribosome and can be modulated by the ribosome itself. Such perturbations are generally believed to be mediated through interactions between the nascent chain and the ribosome surface, but despite recent progress in characterising interactions of unfolded states with the ribosome, and their impact on the initiation of co-translational folding, a complete quantitative analysis of interactions across both folded and unfolded states of a nascent chain has yet to be realised. Here we apply solution-state NMR spectroscopy to measure transverse proton relaxation rates for methyl groups in folded ribosome–nascent chain complexes of the FLN5 filamin domain. We observe substantial increases in relaxation rates for the nascent chain relative to the isolated domain, which can be related to changes in effective rotational correlation times using measurements of relaxation and cross-correlated relaxation in the isolated domain. Using this approach, we can identify interactions between the nascent chain and the ribosome surface, driven predominantly by electrostatics, and by measuring the change in these interactions as the subsequent FLN6 domain emerges, we may deduce their impact on the free energy landscapes associated with the co-translational folding process.

## Abstract 2

NMR measurements of methyl relaxation in translationally-arrested ribosome–nascent chain complexes probe the dynamics of folded nascent polypeptides emerging during biosynthesis and quantify their interaction with the ribosome surface.

## Graphical abstract

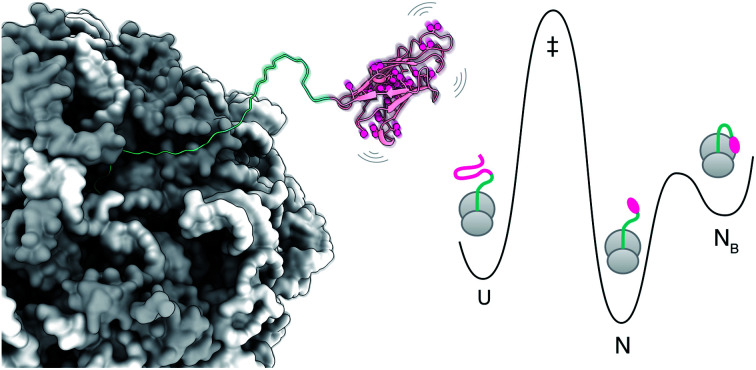

## Introduction

Co-translational folding is a fundamental mechanism for ensuring that nascent polypeptide chains (NCs) efficiently acquire and assemble their correct tertiary and quaternary structures following biosynthesis by the ribosome.^1,2^ It is increasingly apparent that the ribosome itself can play a role in regulating or modulating this process,^3^ and interactions between NCs and the nearby ribosomal surface have been suggested to provide a simple mechanism through which this may be achieved.^4–6^ However, the direct measurement of such intramolecular interactions, involving highly dynamic regions of a 2.3 MDa complex, presents a formidable experimental challenge that has only recently begun to be met.^7^

**Fig. 1:**
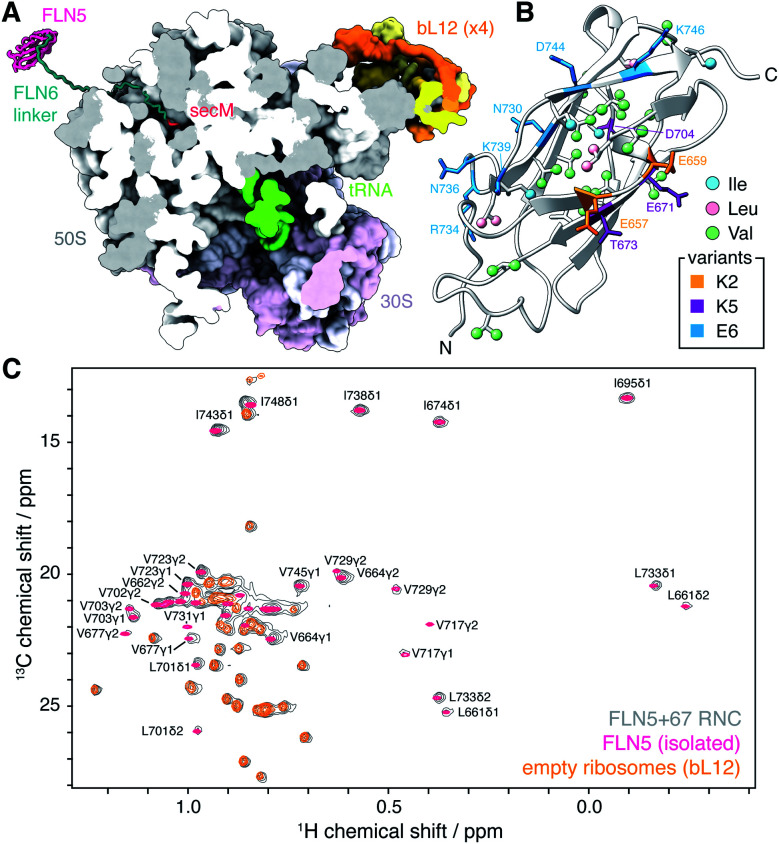
Methyl TROSY NMR spectroscopy of FLN5 RNCs. (A) Modelled structure of an FLN5+67 RNC, comprising the FLN5 domain, FLN6 linker and SecM arrest peptide, emerging from the *E. coli* 70S ribosome (pdb 3j9z^12^). The nascent chain has been built manually in SPDBV^13^ and energy minimised in Foldit.^14^ (B) Cartoon representation of the crystal structure of the FLN5 domain (pdb 1qfh^15^), showing isoleucine, leucine and valine methyls (ball and stick representation, labelled in Fig. S1†), and highlighting residues mutated in charged variants (stick representation). (C) Comparison of ^1^H,^13^C HMQC spectra (298 K, 900 MHz) of ILV-labelled FLN5+67 RNC, isolated FLN5, and unoccupied 70S ribosomes.

We have previously studied the co-translational folding of FLN5, the fifth immunoglobulin domain from the tandem repeat protein filamin, using the SecM arrest peptide to generate translationally-arrested RNCs that are tethered to the peptidyl-transferase center by varying lengths of the subsequent FLN6 domain^8,9^ (Fig. 1A). Through a combination of NMR and biophysical methods, an offset was identified between the emergence of the FLN5 domain from the exit tunnel and the initiation of co-translational folding,^8,10^ due at least in part to strong interactions between the unfolded state and the ribosome surface.^7^ However, an understanding of the dynamics and interactions of both folded and unfolded species is essential to understand the co-translational folding equilibrium fully,^3^ and so in this work we complement measurements of interactions of the unfolded state of FLN5 (ref. ^7^) with a systematic survey of dynamics within folded states.

## Methods

### Sample preparation

Wild-type FLN5 and variants were expressed and purified as previously described.^8^ bL12 was expressed and purified as previously described.^11^ FLN5 RNC samples were expressed and purified following an adaptation of previous protocols:^8,9^*E. coli* BL21 (DE3) cells were adapted progressively into MDG media (100% D_2_O, 2 g L^−1^ d_7_-glucose) and then grown to an OD_600_ of 3 (37 °C, 220 rpm). Cells were resuspended in an equal volume of EM9 media (100% D_2_O, 2 g L^−1^ d_7_-glucose, 80 mg L^−1^ 2-ketobutyric-4-^13^C,3,3-d_2_ acid and 80 mg L^−1^ 2-keto-3-(methyl-^13^C)-butyric-4-^13^C acid). Following 30 min incubation (37 °C, 200 rpm), expression was induced with 1 mM IPTG for 50 min at 37 °C. Cells were then harvested and RNCs purified using previously reported protocols.^7^ Purified RNCs were resuspended in Tico buffer (10 mM HEPES, 30 mM NH_4_Cl, 6 mM MgCl_2_, 0.1% (w/v) EDTA-free protease inhibitor tablet (Roche), pH 7.5), flash frozen and stored at −80 °C.

Unprogrammed [^2^H, ^13^CH_3_-ILV]-labelled 70S ribosomes were prepared from *E. coli* BL21 (DE3) cells grown in MDG media (100% D_2_O, 2 g L^−1^ d_7_-glucose, 80 mg L^−1^ 2-ketobutyric-4-^13^C,3,3-d_2_ acid and 80 mg L^−1^ 2-keto-3-(methyl-^13^C)-butyric-4-^13^C acid) at 37 °C and harvested at an OD_600_ of 3. Ribosomes were purified following the same protocol as RNCs, omitting the metal affinity chromatography step.

### NMR spectroscopy

NMR data were acquired at 298 K on Bruker Avance III HD spectrometers operating at ^1^H Larmor frequencies of 700, 800, 900 and 950 MHz. Data were processed with NMRPipe^16^ and analysed using NMRPipe, NMRFAM-Sparky^17^ and Fuda.^18^

#### Resonance assignment

U-[^13^C,^15^N] labelled FLN5 was prepared at 500 μM in Tico buffer (10 mM HEPES, 30 mM NH_4_Cl, 12 mM MgCl_2_, 1 mM EDTA, pH 7.5), and sidechain resonances were assigned based on a previous backbone assignment^8^ using H(CCO)NH, (H)C(CO)NH, H(C)CH and (H)CCH TOCSY experiments. U-[^13^C,^15^N] labelled bL12 was prepared at 300 μM in Tico buffer, and sidechain resonances were assigned based on a previous backbone assignment^19^ using a 4D HC(CCO)NH TOCSY experiment.

#### Relaxation measurements in isolated FLN5

[^2^H, ^13^CH_3_-ILV]-labelled FLN5 was prepared at *ca.* 100 μM in deuterated Tico buffer (100% D_2_O, d_8_-HEPES, pH* 7.5) containing 0, 40, 50 and 60% (w/w) d_8_-glycerol. ^1^H *R*_2_ relaxation measurements were acquired for each sample using an adapted methyl-SOFAST HMQC experiment (Fig. S3†), with a 300 ms recycle delay and typically 12 relaxation delays. Methyl *S*_axis_^2^*τ*_c_ measurements were obtained from triple quantum build-up and single quantum relaxation measurements.^20^ Calibration curves relating ^1^H *R*_2_ and *S*_axis_^2^*τ*_c_ values were determined using weighted total least squares regression.

#### Relaxation measurements of RNCs

^1^H *R*_2_ relaxation rates were measured for RNC samples using the same pulse sequence as for the isolated domain, with a 300 ms recycle delay and typical sweep widths/acquisition times of 16 ppm/100 ms in the direct dimension and 16 ppm/8.4 ms in the indirect dimension. Recovery delays were 300 ms. Three to five relaxation delays were used, adapted for each RNC sample. RNC samples were defrosted immediately prior to NMR measurements and exchanged into deuterated Tico buffer at final concentrations of *ca.* 10 μM. To monitor sample integrity, a small portion of the sample was incubated at 298 K in parallel with NMR acquisition. Aliquots containing 2 pmol of RNC were periodically flash frozen for analysis of tRNA-bound fractions by anti-His western blotting. Nascent chain attachment was also monitored using ^13^C-filtered ^1^H STE diffusion experiments,^21^ which were interleaved at *ca.* 3 h intervals throughout data acquisition, with a diffusion delay of 50 ms, and 4 ms bipolar smoothed-square gradient pulses. Diffusion coefficients were typically measured using three well resolved methyl resonances originating from the nascent chain, but where this was not possible due to low signal to noise (+47, K2 and K5 variants), bL12 resonances were instead used to monitor sample integrity. K5 RNC samples were treated with 60 μg ml^−1^ RNase A to release the NC following acquisition.

#### Estimation of ^1^H *R*_2_ rates in the K5 RNC

Lower bounds for ^1^H *R*_2_ rates in the K5 RNC were estimated by comparison of the spectrum noise level (as an upper limit on peak intensities), *σ*_K5_, relative to peak intensities in the wild-type FLN5+67 spectrum, *I*_WT_ (normalised by the total number of scans and nascent chain concentration as assessed by immunoblotting^9^). ^1^H and ^13^C linewidths for the wild-type NC (lw_H_ and lw_C_, in Hz) were determined using NMRPipe. Accounting for the effects of line widths and relaxation during the pulse sequence on the intensity of cross-peaks, the relative intensity in the two experiments is:^22^1
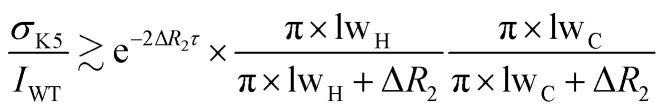
where Δ*R*_2_ = *R*^K5^_2_−*R*^wt^_2_ and *τ* = 1/2*J*_CH_ = 4 ms. From this expression, lower bounds for *R*^K5^_2_ = *R*^wt^_2_ + Δ*R*_2_ could be determined. To reduce the sensitivity to changes in the ^13^C relaxation rate, which we assume here to be comparable to the change in the ^1^H relaxation rate, both spectra were processed with a strong 150 Hz exponential window function in the indirect dimension (and a 30 Hz exponential window function in the direct dimension).

## Results and discussion

We have previously found that structured FLN5 RNCs were most effectively observed using deuteration and selective ^13^CH_3_ labelling of isoleucine C^δ^ methyl groups.^8^ However, as this provides rather sparse coverage across the domain, to enable a more detailed structural analysis here we have expanded our labelling approach to include leucine and valine methyl groups, which are widely distributed throughout the domain (Fig. 1B and S1†). We selected the FLN5+67 RNC (Fig. 1A) as a starting point for our study, as this long linker sequence, comprising a fragment of the subsequent FLN6 domain together with the SecM arrest peptide, ensures that the FLN5 domain has emerged completely from the ribosome exit tunnel and can fold to a native-like state.^8^ The resulting ^1^H,^13^C correlation spectrum is shown in Fig. 1C, and from this approximately twenty FLN5 resonances can be resolved unambiguously. These resonances overlay closely with those of isolated FLN5, indicating that there are no structural perturbations within the folded nascent chain. Additional resonances are also observed in spectra of unprogrammed ILV-labelled ribosomes, and can be attributed to the mobile C-terminal domain of the bL12 stalk protein, previously observed using ^15^N-based methods^11,22,23^ (Fig. S2†). Despite the heightened sensitivity of the labelling scheme and methyl TROSY experiment, no additional ribosomal signals were detected in these measurements.

**Fig. 2:**
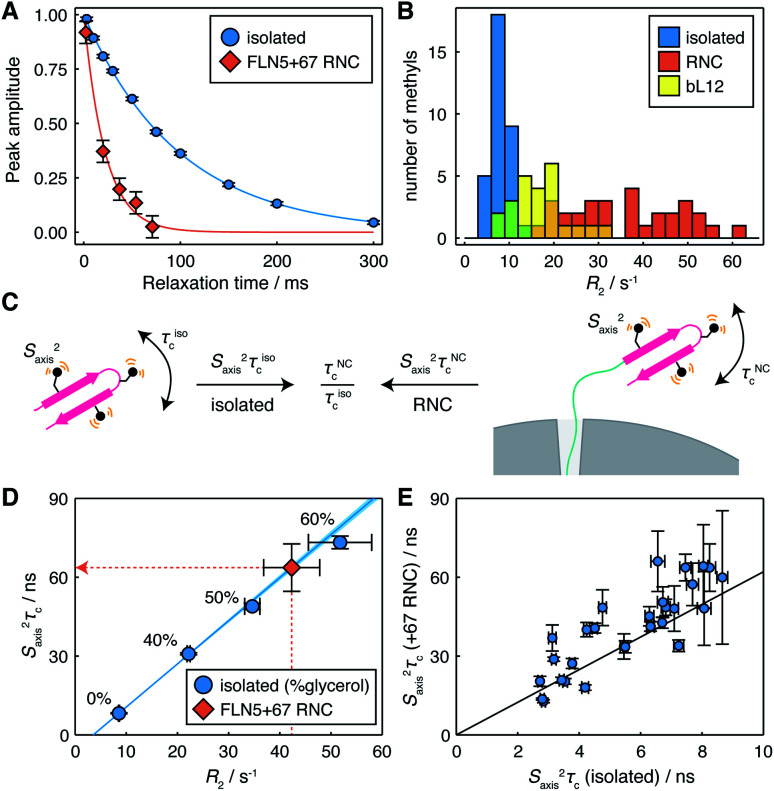
Measurement of methyl ^1^H relaxation and analysis of mobility for the FLN5+67 RNC. (A) ^1^H *R*_2_ measurements for the representative residue L661^δ2^ in isolated FLN5 and FLN5+67 RNC (298 K, 950 MHz). Measurements for other residues are plotted in Fig. S5.† (B) Distribution of *R*_2_ measurements in isolated FLN5, FLN5+67 RNC and the bL12 C-terminal domain (298 K, 950 MHz). (C) Illustration of the cancellation of *S*_axis_^2^ terms through the comparison of dynamics in isolated FLN5 and FLN5 RNCs. (D) Correlation between ^1^H *R*_2_ rates and *S*_axis_^2^*τ*_c_ values measured for L661^δ2^ in isolated FLN5 at varying concentrations of d_8_-glycerol, also illustrating the conversion from ^1^H *R*_2_ rates to *S*_axis_^2^*τ*_c_ values for the FLN5+67 RNC. (E) Correlation between *S*_axis_^2^*τ*_c_ values measured for methyl groups in isolated FLN5, and *S*_axis_^2^*τ*_c_ values determined from ^1^H *R*_2_ rates for the FLN5+67 RNC. Error bars show standard errors propagated from *R*_2_ or cross-correlated relaxation measurements.

To investigate the mobility of the NC we then acquired ^1^H *R*_2_ relaxation measurements, using an adapted methyl-SOFAST HMQC^24^ incorporating a Hahn echo and a filter for slowly relaxing inner transitions (Fig. S3†). These measurements were acquired in *ca.* 3 h blocks interleaved with control measurements and parallel biochemical assays to monitor sample integrity (Fig. S4†). The measured relaxation data fitted well to single exponential decays (Fig. 2A and S5†), and resonances in the FLN5+67 RNC showed significantly higher relaxation rates (36 ± 12 s^−1^, mean ± s.d.) than in the isolated protein (8.4 ± 2.5 s^−1^, mean ± s.d.) (Fig. 2B), indicating that the mobility of the NC is reduced. As the differences in *R*_2_ relaxation rates between the isolated domain and RNC varied significantly between methyl groups, we can exclude a constant lifetime line broadening effect due to interactions with the ribosome surface in slow chemical exchange.^25^ Instead, changes in relaxation rates are consistent either with a reduction in the rotational diffusion of the domain itself, or with transferred relaxation from the ribosome occurring in the fast chemical exchange limit. Relaxation rates for the FLN5 domain were also much greater than those of the bL12 CTD (Fig. 2B, 17 ± 6 s^−1^, mean ± s.d.). As the effective rotational correlation time, *τ*_c_, of the bL12 CTD has previously been determined as *ca.* 14 ns,^22^ this provides an approximate lower bound for the mobility of the NC.

The mobility of methyl groups within folded domains can be described by the product *S*_axis_^2^*τ*_c_, where *S*_axis_^2^ is an order parameter for the three-fold methyl symmetry axis, representing motion on a ps–ns timescale, and *τ*_c_ is an effective rotational correlation time, which may depend on the orientation of the methyl symmetry axis relative to the rotational diffusion tensor, as well as transient interactions with the ribosome surface (Fig. 2C). However, each methyl *R*_2_ relaxation rate, measured here, will depend differently on changes in *τ*_c_, due to a combination of effects: various dipolar relaxation pathways involving spectral densities at multiple frequencies, and chemical exchange. Taking an empirical approach, we therefore constructed calibration curves for each methyl group using the isolated protein at varying concentrations of glycerol in order to relate observed *R*_2_ rates (Table S2†) to the effective mobility, *S*_axis_^2^*τ*_c_ (Table S3†), obtained using measurements of cross-correlated relaxation^20^ (Fig. 2D and S6†).

Using these calibration curves, measured *R*_2_ rates for RNCs (Table S4†) could be converted to *S*_axis_^2^*τ*_c_ values (Fig. 2D and Table S5†) and subsequently compared with the isolated protein (Fig. 2E). As *S*_axis_^2^ order parameters are a local property of the domain structure, and given that methyl chemical shifts, and ^13^C chemical shifts in particular, are highly sensitive to changes in structure and rotamer distribution,^26^ we argue that it is reasonable to assume that they do not change significantly between the isolated protein and the NC. Therefore, changes in *S*_axis_^2^*τ*_c_ between a NC and the isolated protein may be interpreted purely in terms of the relative change in the effective correlation times, *τ*^NC^_c_/*τ*^iso^_c_ (Fig. 2C).

In the case of the FLN5+67 RNC, we observe a strong correlation (*r* = 0.85 ± 0.06) between *S*_axis_^2^*τ*_c_ values in isolated FLN5 and in the NC (Fig. 2E). Linear regression analysis indicates a *ca.* six-fold reduction in mobility of the NC (*τ*^NC^_c_/*τ*^iso^_c_ = 6.2 ± 0.3, Table 1), in good agreement with an earlier estimate of 5.2 obtained from cross-correlated relaxation measurements of three isoleucine resonances.^27^ The strong correlation indicates that the rotational diffusion tensor is being scaled rather than deformed or rotated by the presence of the ribosome. This is most consistent with transferred relaxation due to transient interactions with the surface, although the deviations may indicate some additional degree of anisotropic motion due to the tethering of the domain by the FLN6 linker, giving an orientational dependence to the local *τ*_c_ of methyl groups. These results also indicate that broadened resonances previously reported for a uniformly ^1^H,^13^C-labelled FLN5 RNC^28^ correspond closely with those exhibiting high *S*_axis_^2^*τ*_c_ values in the isolated domain (Fig. S6†), *i.e.* strongly ordered resonances are naturally relaxed more efficiently, and highlights the benefit of the selective methyl labelling scheme that we have applied in the present work.

**Table 1:** FLN5 rotational correlation times determined for the isolated domain and within RNCs

Sample	Relative *τ*_c_	*τ*_c_/ns
Isolated FLN5 (0% glycerol)	1	7.7 (ref. ^29^)
Isolated FLN5 (40% glycerol)	3.4 ± 0.1	26.2 ± 0.8
Isolated FLN5 (50% glycerol)	5.1 ± 0.1	39.3 ± 0.8
Isolated FLN5 (60% glycerol)	7.5 ± 0.2	57.8 ± 1.5
wt FLN5 +47 RNC	10.6 ± 0.9	82 ± 7
wt FLN5 +57 RNC	10.4 ± 0.6	80 ± 5
wt FLN5 +67 RNC	6.2 ± 0.3	48 ± 2
wt FLN5 +110 RNC	4.2 ± 0.2	32 ± 2
wt FLN5 +67 RNC, GS linker	6.6 ± 0.4	51 ± 3
E6 FLN5 +67 RNC	5.6 ± 0.4	43 ± 3
K2 FLN5 +67 RNC	15.6 ± 1.5	120 ± 12
K5 FLN5 +67 RNC	≳100	≳770
70S ribosome	430	3600 (ref. ^30^)

**Fig. 3:**
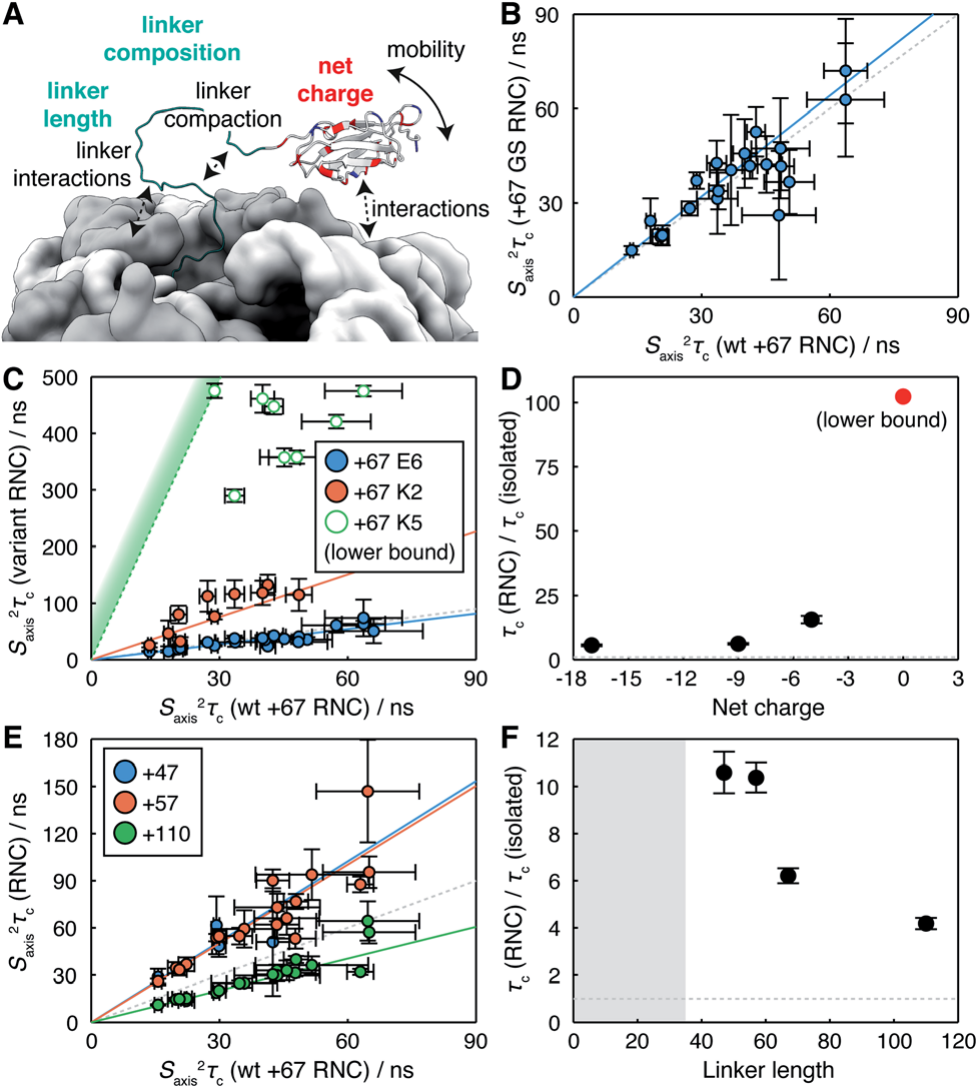
Analysis of the determinants of reduced NC mobility. (A) Illustration of the potential influences on NC mobility explored within this work. (B) Correlation between *S*_axis_^2^*τ*_c_ values determined from ^1^H *R*_2_ rates for FLN5+67 RNCs comprising wild-type FLN6 and poly-GS linker sequences. (C) Correlation between *S*_axis_^2^*τ*_c_ values determined from ^1^H *R*_2_ rates for FLN5+67 charge variants and wild-type FLN5+67. Open circles for the K5 variant indicate approximate lower bounds determined from an analysis of signal intensity. (D) Changes in rotational correlation times for FLN5+67 RNC charge variants, relative to the isolated domain. (E) Correlation between *S*_axis_^2^*τ*_c_ values determined from ^1^H *R*_2_ rates for FLN5 RNCs as a function of FLN6 linker length, relative to wild-type FLN5+67. (F) Changes in rotational correlation times for FLN5 RNCs, relative to the isolated domain, as a function of linker length. Shading indicates the approximate extent of polypeptide enclosed within the exit tunnel.^8^

Having established an effective measurement strategy, we sought to investigate systematically how basic physical properties of the NC affect its mobility and interactions with the ribosome surface. In particular, by comparison of engineered RNC variants (Table S1†) we sought to distinguish unambiguously between changes in the mobility of the NC due to tethering, interactions of the FLN5 domain with the ribosome surface, and intra- or inter-molecular interactions of the FLN6 linker itself (Fig. 3A).

We first explored the effect of substituting the FLN6 linker for a poly-GS sequence that is neutral, non-hydrophobic, disordered, and that has previously been shown not to alter the onset of co-translational folding,^8^ or to perturb interactions of the unfolded state.^7^^1^H *R*_2_ measurements were acquired for a FLN5 RNC containing a 67 residue GS linker (comprising 48 GS residues and the 19 residue SecM arrest peptide), and no significant differences were found with respect to the WT FLN5+67 RNC. This is presented in terms of estimated *S*_axis_^2^*τ*_c_ values in Fig. 3B, and a regression analysis indicates a relative mobility *τ*^GS^_c_/*τ*^WT^_c_ = 1.07 ± 0.03 (*r* = 0.85 ± 0.13). Thus, we conclude that neither the residual structure, compaction or interactions of the linker region are responsible for the reduced mobility of the FLN5 domain within RNCs.

We next examined the effect of varying the charge of the FLN5 domain (net charge *Z* = −9) on electrostatic interactions with the predominantly negatively charged ribosome surface.^31^ We have previously introduced the E6 variant for the analysis of interactions of the unfolded state,^7^ in which six surface residues were substituted with negatively charged glutamate residues (N730E/R734E/N736E/K739E/D7444E/K746E, *Z* = −17, Fig. 1C). Expanding on this, here we have generated two additional variants, K2 (E657K/E659K, *Z* = −5) and K5 (K2/E671K/T673K/D704K, *Z* = 0), in which a second cluster of surface residues were mutated to positively charged lysine residues (Fig. 1C). As the K5 variant was already found to interact strongly (see below), no further positively charged variants were generated. All variants remained fully folded under the conditions used in this work, and we observed very limited methyl chemical shift perturbations (Fig. S7†), indicating that the core structure of the variants was not affected by the surface mutations. On this basis we have used the same calibration curves for the interpretation of *R*_2_ measurements as for WT FLN5 (Fig. 2D).

RNC samples of the E6, K2 and K5 FLN5 variants were prepared using a constant linker length of 67 for comparison with the WT observations above. *R*_2_ measurements were successfully acquired for the E6 and K2 variants (Table S4†), although as a consequence of more rapid relaxation the intensity of resonances in the K2 variant were reduced *ca.* 4-fold relative to the WT. No NC resonances could be detected from the K5 variant. However, translational diffusion measurements of bL12 resonances confirmed the integrity of the core ribosome particle, and the presence of the NC was confirmed both by immunoblotting, and by observation of the released NC following treatment with RNase (Fig. S3†). In line with changes in intensity and relaxation rates observed between the WT and K2 variants, we therefore conclude that the mobility of the K5 NC is reduced further, likely due to stronger interactions following the elimination of the net negative charge.

*S*_axis_^2^ *τ*_c_ values estimated from measured relaxation rates are plotted in Fig. 3C. Strong linear correlations were observed for both E6 and K2 variants (*r* = 0.89 ± 0.10 and 0.85 ± 0.12 respectively), from which the relative changes in mobility may be determined. As discussed earlier for the WT RNC, such linear correlations are consistent with transferred relaxation from a bound state, and the reduction in mobility as the net charge on the NC decreases indicates the role of electrostatic interactions between positively charged patches on the NC and the negatively charged ribosome surface. However, given that the WT and E6 variant also exhibit similar behaviour (Fig. 3C), the net charge alone is clearly insufficient to fully characterise interactions, indicating that factors such as the distribution of charges across the surface of the domain may also be relevant. For the K5 variant, lower limits for relaxation rates and hence *S*_axis_^2^*τ*_c_ could be estimated from the spectrum noise level. The strongest constraint on NC mobility was provided by the I743 resonance, the intensity of which was less than 4% of that of the WT RNC, from which we estimate that the *R*_2_ ≳480 s^−1^ and therefore *τ*^K5^_c_/*τ*^iso^_c_ ≳ 100 (Fig. 3D).

Lastly, we have probed the effect of varying the length of the subsequent FLN6 tether, mimicking the progressive emergence of the domain from the ribosome exit tunnel during translation. This has previously been found to modulate interactions between the unfolded state and the ribosome surface by varying the effective concentration of binding sites on the ribosome surface.^6^ Here, four linker lengths were examined, from 47 to 110 residues (of which *ca.* 30–35 residues are enclosed within the exit tunnel^7^) (Fig. 3E). An increase in mobility was observed with increasing chain lengths (Fig. 3F), consistent with a decrease in the effective concentration of interaction sites on the ribosome surface.

Collectively, our observations of the effects of net charge, linker length and linker composition on the mobility of the FLN5 domain can be accounted for most simply by the transient interaction of the FLN5 domain with the ribosome surface. Given that the (isotropic) rotational correlation time of the isolated domain (in D_2_O at 298 K) is 7.7 ns,^29^ and the ribosome itself is *ca.* 3.6 μs,^30^ complete binding would correspond to a change in effective correlation time τ^ribo^_c_/τ^iso^_c_ ≈ 430. Thus, for the wild-type RNC from linker lengths of 47 to 110 (Fig. 3F), we estimate that the fraction of bound NC decreases from *ca.* 2.2% to 0.9%.

**Fig. 4:**
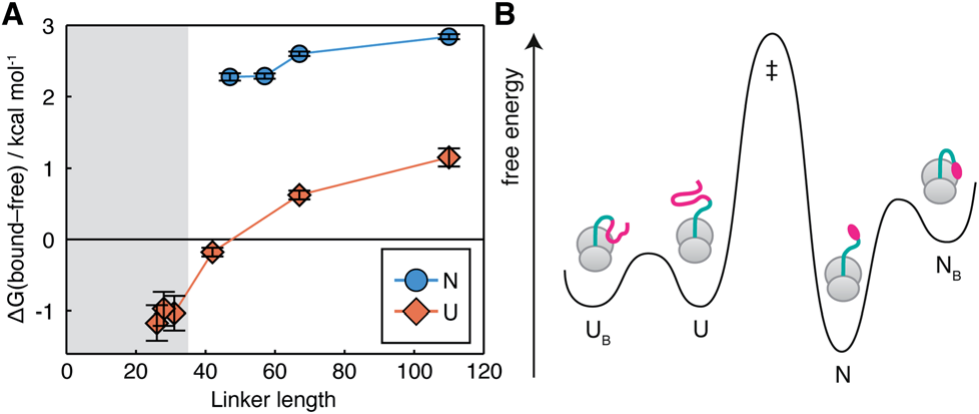
Impact of NC–ribosome interactions on energetics of co-translational folding. (A) Free energy of FLN5–ribosome interactions for folded (this work) and unfolded^7^ states as a function of polypeptide chain length. Shading indicates the approximate extent of polypeptide enclosed within the exit tunnel.^8^ (B) Free energy diagram for the FLN5+47 RNC, combining measurements of native state interactions in this work with measurements of folding energetics^10^ and unfolded state interactions.^7^ Barrier heights are illustrative only.

The interactions we observe here can be compared with the much stronger interaction of a C-terminal segment within unfolded NCs, which has a bound population of up to 90% at short tether lengths.^7^ This is plotted here in terms of the free energy of interaction, 
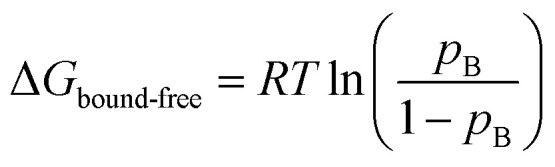
 (Fig. 4A). The dependence of Δ*G*_bound-free_ on linker length is similar for observed lengths of native and unfolded states, indicating that the changing linker modulates the effective concentration of the ribosome surface to a similar extent. However, interactions of the unfolded state are approximately 2 kcal mol^−1^ stronger than for the native state, corresponding to a *ca.* 30-fold difference in association constant. We may combine these data with previous measurements of co-translational folding energetics,^10^ to construct a free energy diagram illustrating for the first time the impact of interactions of both folded and unfolded states on the co-translational folding of a NC (Fig. 4B).

We have previously shown that the effect of an interaction on the net energetics of folding is:^7^

2 ΔΔ*G*_folding_ = ±*RT* ln(1 − *p*_B_)

where the sign depends on whether the interaction involves the folded or unfolded state. This indicates that, in this case at least, the weak interactions of the native state, corresponding to a sparsely populated, high energy bound state (Fig. 4B), do not significantly perturb the co-translational folding process. However, we have also found that interactions of both native and unfolded states can be strongly and differentially modulated by changing electrostatics: the E6 variant destabilizes interactions of the unfolded state by 1.9 ± 0.1 kcal mol^−1^,^7^ without perturbing the interactions of the native state; but equally, interactions of the native state can be increased to at least *ca.* 25% within the K5 variant (Fig. 3C and D). Therefore, these results indicate that simple physicochemical properties such as net charge are not by themselves sufficient to understand or predict the strength of interactions: folding and local structure also clearly play an important role.

## Conclusions

The ribosome has now been shown to perturb the co-translational folding of many different proteins, generally leading to a reduction in stability relative to the isolated domain.^4,5,8,32–34^ This is usually ascribed to the effect of interactions, although given the diversity of the proteome, such interactions are likely to be variable in strength and significance. Ultimately, the ability to predict the strength of these interactions would be valuable in better understanding the general mechanisms of co-translational folding. The measurements we have undertaken in this work, together with analogous measurements of the unfolded state,^7^ provide initial steps in this direction. By determining the first complete description of interactions for both sides of the folding equilibrium, we may begin to progress studies of co-translational folding towards a quantitative standard comparable to that achieved for the reversible folding of isolated domains.

## Data availability

Relaxation and cross-correlation relaxation datasets supporting this article have been uploaded as part of the ESI.† Side-chain resonance assignments for FLN5 and bL12 have been deposited in the BMRB under accession numbers 51075 and 51076.

## Author contributions

Conceptualization: CAW, JC. Funding acquisition: JC. Investigation: CB, CAW. Methodology: CAW, TW. Supervision: CAW, AMEC, LDC, JC. Writing – original draft: CB, CAW, JC. Writing – review & editing: all authors.

## Conflicts of interest

There are no conflicts to declare.

## Supplementary Material

SC-012-D1SC04313G-s001
