# Supplementary material for: Nascent chain dynamics and ribosome interactions within folded ribosome–nascent chain complexes observed by NMR spectroscopy
Source: Chem Sci. 2021 Sep 9;12(39):13120–6. doi: 10.1039/d1sc04313g (PMC8513902; doi:10.1039/d1sc04313g)
Supplement: SC-012-D1SC04313G-s001 [file SC-012-D1SC04313G-s001.pdf]

Electronic Supplementary Material (ESI) for Chemical Science.
This journal is © The Royal Society of Chemistry 2021

Supplementary Information

Nascent chain dynamics and ribosome interactions within folded ribosome–nascent chain complexes observed by NMR spectroscopy

Charles Burridge, Christopher A. Waudby, Tomasz Włodarski, Anaïs M. E. Cassaignau, Lisa D. Cabrita and John Christodoulou**

Department of Structural and Molecular Biology, University College London, London WC1E 6BT, UK.

* Email: c.waudby@ucl.ac.uk, j.christodoulou@ucl.ac.uk

Table of Contents

Supplementary Figures

Fig. S1 Annotated crystal structure of FLN5

Fig. S2 ^{1}H,^{13}C HMQC spectra of unoccupied 70S ribosomes and unlabeled isolated bL12

Fig. S3 Pulse program for the measurement of methyl ^{1}H R_2 relaxation rates

Fig. S4 Time courses for quality control of RNC samples

Fig. S5 Methyl ^{1}H R_2 measurements for isolated FLN5 and FLN5+67 RNC

Fig. S6 Correlation between ^{1}H R_2 rates and $S^2_{axis}\tau_c$ values in isolated FLN5 vs glycerol

Fig. S7 Correlation between $S^2_{axis}\tau_c$ values in isolated FLN5 and FLN5+67 RNC

Fig. S8 Comparison of ^{1}H,^{13}C HMQC spectra of FLN5 wild-type, K2 and K5 variants.

Supplementary Tables

Table S1 Protein sequences for nascent chains used in this study

Table S2 Methyl ^{1}H R_2 for isolated FLN5 vs glycerol concentration

Table S3 Methyl $S^2_{axis}\tau_c$ values for isolated FLN5 vs glycerol concentration

Table S4 Methyl ^{1}H R_2 measurements for RNCs

Table S5 Calculated methyl $S^2_{axis}\tau_c$ values for RNCs

References

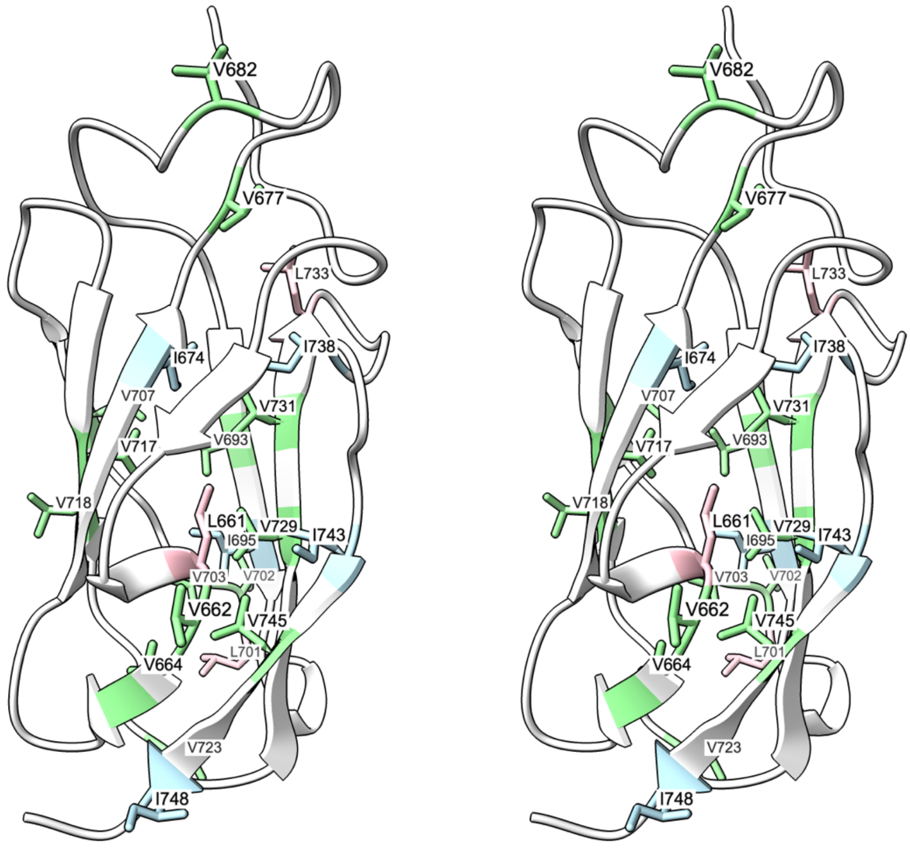

Supplementary Figure 1: Stereoview cartoon representation of the FLN5 crystal structure (pdb: 1qfh) showing the location of isoleucine, leucine and valine residues.

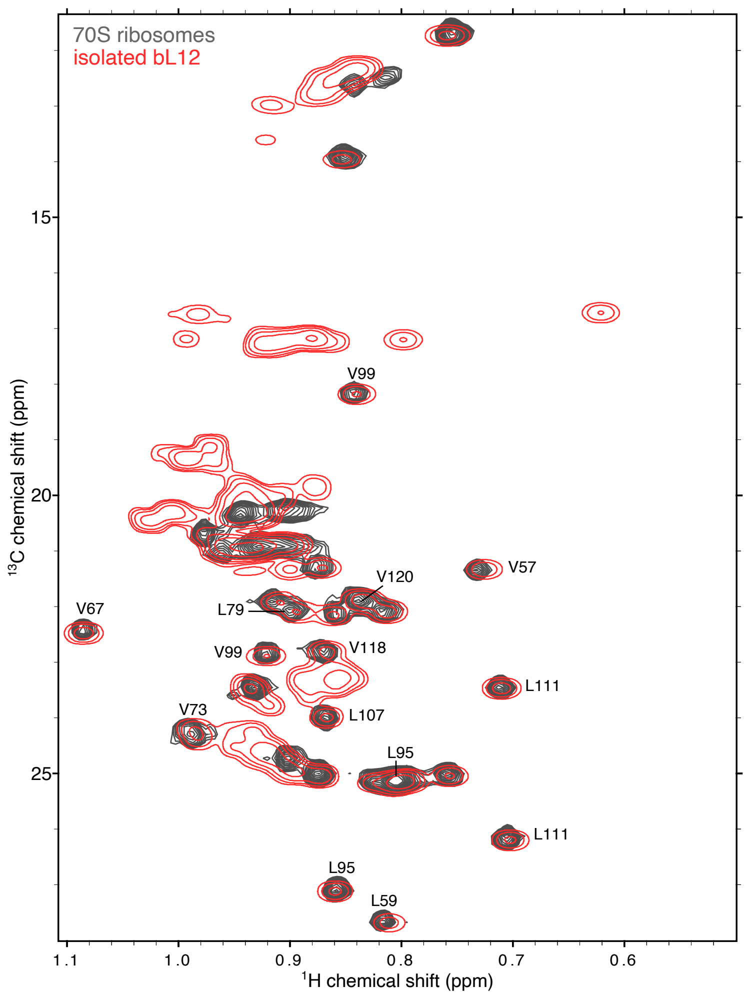

Supplementary Figure 2: ^{1}H,^{13}C HMQC spectra of ILV-labelled unoccupied 70S ribosomes (black, 298 K, 950 MHz) and unlabeled isolated bL12 (red, 298 K, 700 MHz).

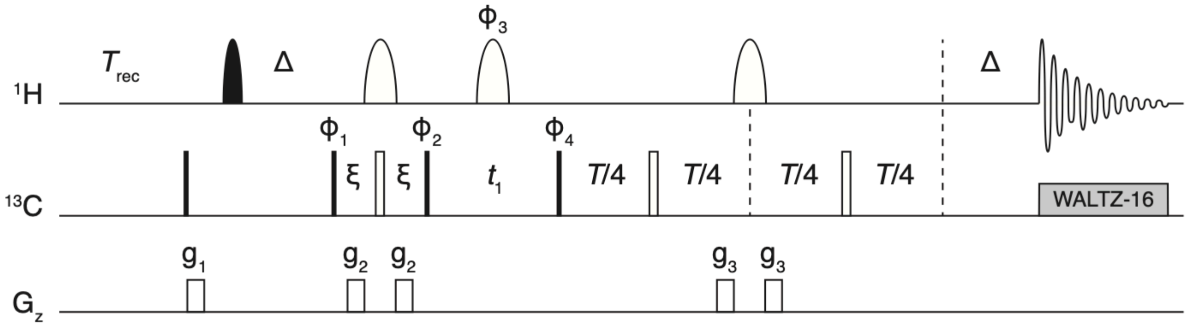

Supplementary Figure 3: Pulse program for the measurement of methyl ^{1}H R_2 relaxation rates, based on a methyl SOFAST-HMQC experiment incorporating a filter for slowly relaxing inner transitions. T is the relaxation time, $\Delta = 1/2J_{CH} = 4$ ms, and $\xi = 1/8J_{CH} = 1$ ms. ^{1}H shaped pulses were applied at 0.5 ppm; the solid pulse represents a 120º Pc9 excitation pulse (1120 µs at 950 MHz) and the hollow pulse represents a 180º Rsnob pulse (564 µs at 950 MHz). Phase cycles: ϕ_1 = 0°,180°; ϕ_2 = $(0°)_2$, $(180°)_2$; ϕ_3 = $(0°)_8$, $(120°)_8$, $(240°)_8$ ϕ_4 = $(0°)_4$, $(180°)_4$ and ϕ_{rx} = 0°, 180°, 0°, 180°, 180°, 0°, 180°, 0°, 120°, 300°, 120°, 300°, 300°, 120°, 300°, 120°, 240°, 60°, 240°, 60°, 60°, 240°, 60°, 240°, with ϕ_4 incremented for States-TPPI quadrature detection. Gradient length, shape and power are: g_1 = 1 ms (SMSQ10.100, 31%); g_2 = 50 µs (SINE.10, –40%); g_3 = 100 µs (SINE.10, 11%).

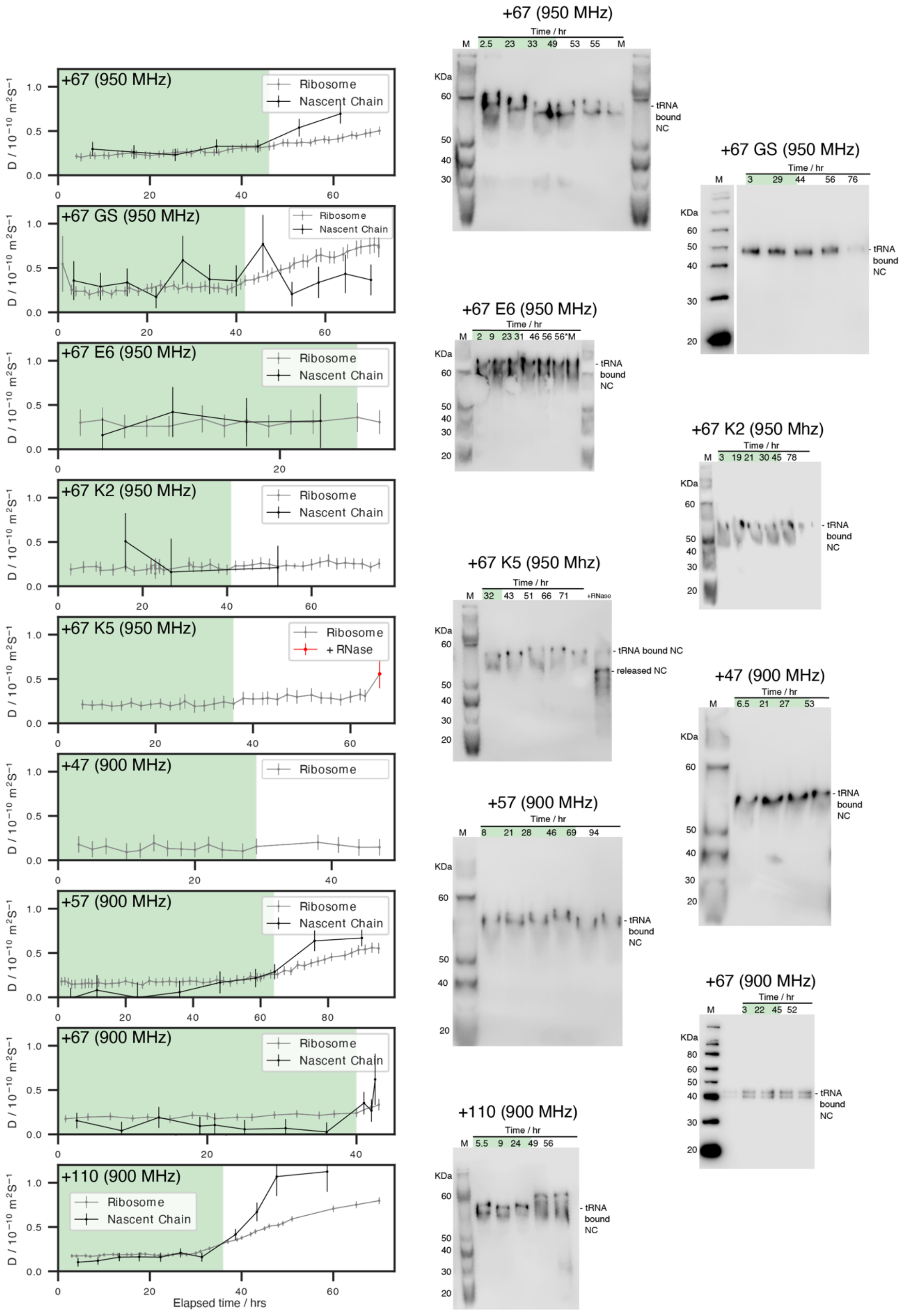

Supplementary Figure 4: Time courses for quality control of RNC samples. (**A**) Time course of nascent chain (I674, I738, I695 methyl resonances) and bL12 diffusion coefficients during data acquisition, shaded green area indicates measurement time used for analysis (**B**) Time course of nascent chain attachment by western blot (anti-histidine), using 2 pmol RNC aliquots taken periodically from a sample incubated in parallel with NMR data acquisition. The tRNA-bound nascent chain and released NC is indicated.

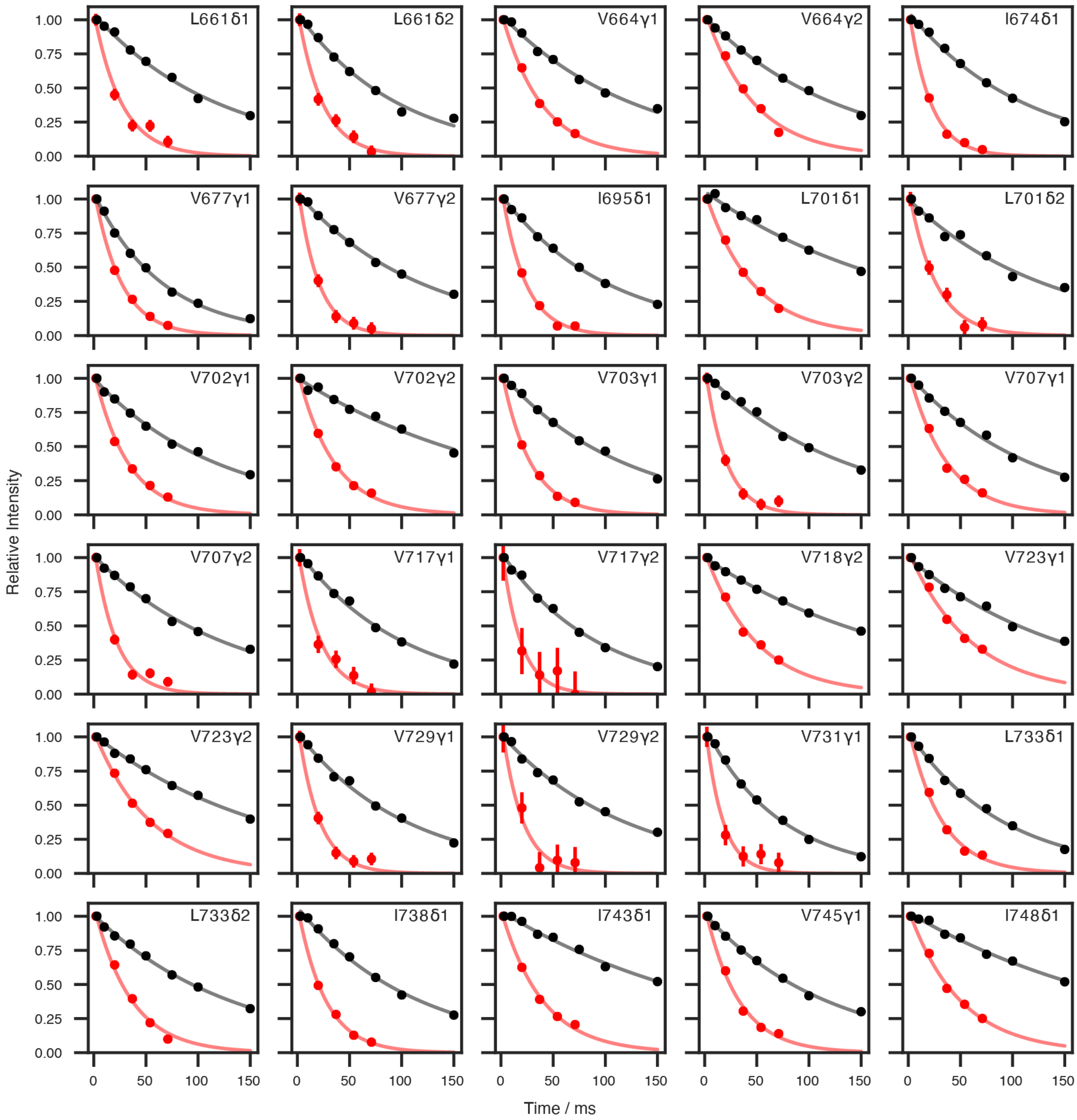

Supplementary Figure 5: Methyl ^{1}H R_2 measurements for isolated FLN5 (black) and FLN5+67 RNC (red) (298 K, 950 MHz).

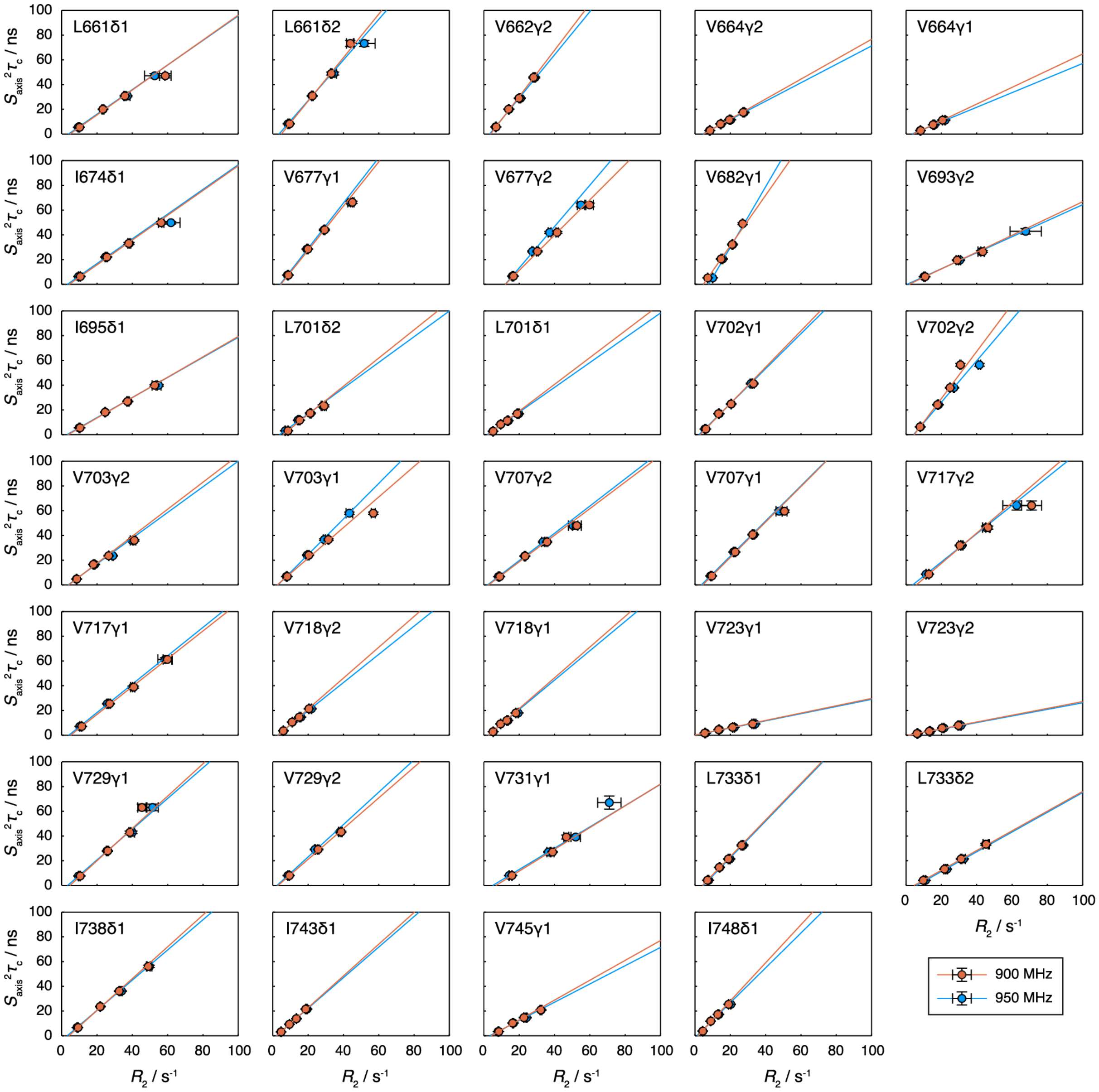

Supplementary Figure 6: Correlation between ^{1}H R_2 rates and $S_{axis}^2\tau_c$ values for isoleucine, leucine and valine methyls in isolated FLN5 at varying concentrations of d_8-glycerol at 298 K, with ^{1}H R_2 rates acquired at 900 MHz (orange) or 950 MHz (blue).

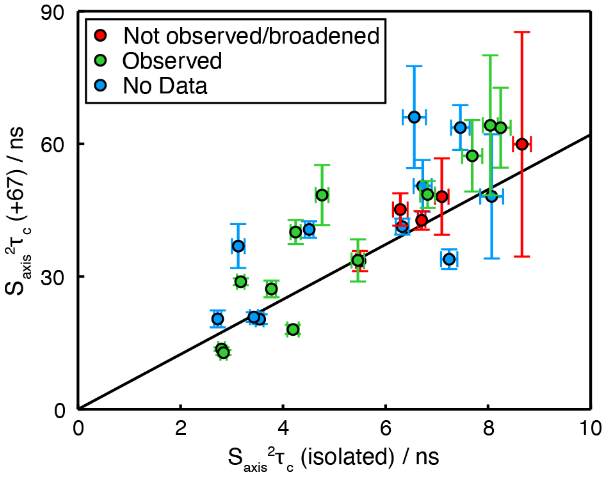

Supplementary Figure 7: Correlation between $S^2_{axis}\tau_c$ values measured for isolated FLN5, and $S^2_{axis}\tau_c$ values determined from ^1H R_2 rates for the FLN5+67 RNC as shown in Fig. 2D, colored by observability of resonances previously reported for a uniformly ^1H,^{13}C-labelled FLN5 RNC[1].

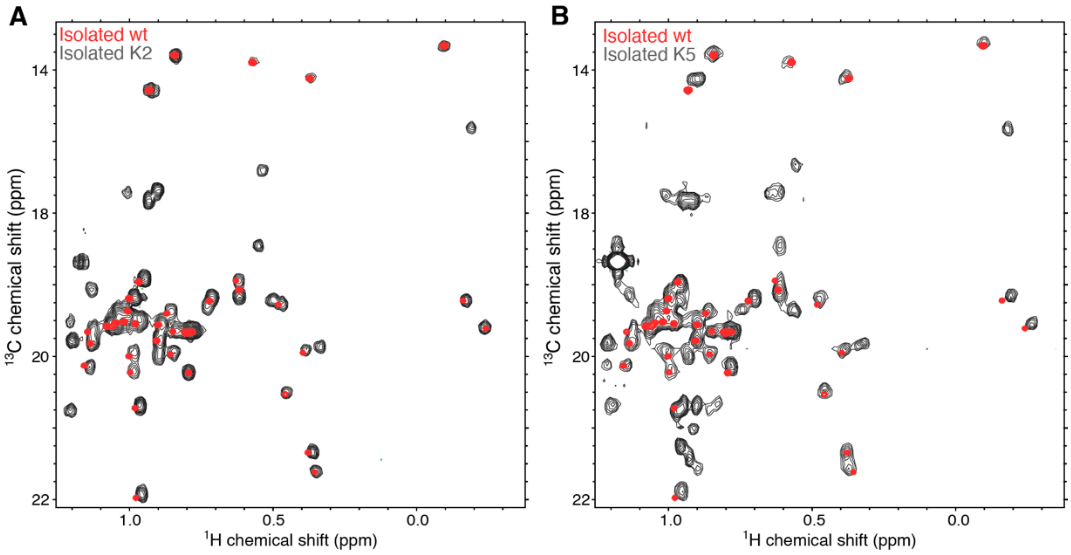

Supplementary Figure 8: Comparison of ^{1}H,^{13}C HMQC spectra of ILV-labelled FLN5 (298 K, 950 MHz) with isolated unlabeled (**A**) K2 FLN and (**B**) K5 FLN (298 K, 500 MHz).

Construct	Sequence
+47	MHHHHHHASKPAPSAEHSYAEGEGLVKVFDNAPAEFTIFAVDTKGVARTDGGDPFEVAINGPDGLVVDAKVTDNNDGTYG<br>VVYDAPVEGNYNVNVTLRGNPIKNMPIDVKCIEGANGEDSSFGSFTFTVAAKNKKGEVKTYGELFSTPVWIWWWPRIRGPP
+57	MHHHHHHASKPAPSAEHSYAEGEGLVKVFDNAPAEFTIFAVDTKGVARTDGGDPFEVAINGPDGLVVDAKVTDNNDGTYG<br>VVYDAPVEGNYNVNVTLRGNPIKNMPIDVKCIEGANGEDSSFGSFTFTVAAKNKKGEVKTYGGDKFEVSITGELFSTPVW<br>IWWWPRIRGPP
+67	MHHHHHHASKPAPSAEHSYAEGEGLVKVFDNAPAEFTIFAVDTKGVARTDGGDPFEVAINGPDGLVVDAKVTDNNDGTYG<br>VVYDAPVEGNYNVNVTLRGNPIKNMPIDVKCIEGANGEDSSFGSFTFTVAAKNKKGEVKTYGGDKFEVSITGPAEEITLD<br>AIELFSTPVWIWWWPRIRGPP
+110	MHHHHHHASKPAPSAEHSYAEGEGLVKVFDNAPAEFTIFAVDTKGVARTDGGDPFEVAINGPDGLVVDAKVTDNNDGTYG<br>VVYDAPVEGNYNVNVTLRGNPIKNMPIDVKCIEGANGEDSSFGSFTFTVAAKNKKGEVKTYGGDKFEVSITGPAEEITLD<br>AIDNQDGTYTAAYSLVGNGRFSTGVKLNGKHIEGSPFKQVLGNTSEFFSTPVWIWWWPRIRGPP
+67 GS	MHHHHHHASKPAPSAEHSYAEGEGLVKVFDNAPAEFTIFAVDTKGVARTDGGDPFEVAINGPDGLVVDAKVTDNNDGTYG<br>VVYDAPVEGNYNVNVTLRGNPIKNMPIDVKCIEGGGGSGGGGSGGSGGGSGGGGSGGGSGGSGGGSGGGGSGGGSGGGSG<br>GGELFSTPVWIWWWPRIRGPP
+67 K2	MHHHHHHASKPAPSAEHSYAKGKGLVKVFDNAPAEFTIFAVDTKGVARTDGGDPFEVAINGPDGLVVDAKVTDNNDGTYG<br>VVYDAPVEGNYNVNVTLRGNPIKNMPIDVKCIEGANGEDSSFGSFTFTVAAKNKKGEVKTYGGDKFEVSITGPAEEITLD<br>AIELFSTPVWIWWWPRIRGPP
+67 K5	MHHHHHHASKPAPSAEHSYAKGKGLVKVFDNAPAKFKIFAVDTKGVARTDGGDPFEVAINGPDGLVVKAKVTDNNDGTYG<br>VVYDAPVEGNYNVNVTLRGNPIKNMPIDVKCIEGANGEDSSFGSFTFTVAAKNKKGEVKTYGGDKFEVSITGPAEEITLD<br>AIELFSTPVWIWWWPRIRGPP
+67 E6	MHHHHHHASKPAPSAEHSYAEGEGLVKVFDNAPAEFTIFAVDTKGVARTDGGDPFEVAINGPDGLVVDAKVTDNNDGTYG<br>VVYDAPVEGNYNVEVTLEGEPIENMPIEVECIEGANGEDSSFGSFTFTVAAKNKKGEVKTYGGDKFEVSITGPAEEITLD<br>AIELFSTPVWIWWWPRIRGPP

Supplementary Table 1: Protein sequences for nascent chains used in this study.

Methyl	0% 950 MHz	0% 900 MHz	40% 950 MHz	40% 900 MHz	50% 950 MHz	50% 900 MHz	60% 950 MHz	60% 900 MHz
L661δ1	8.6 ± 0.1	9.5 ± 0.1	22.2 ± 0.3	22.4 ± 0.4	34 ± 1	33.1 ± 0.5	51 ± 6	43 ± 2
L661δ2	9.3 ± 0.1	10.2 ± 0.1	23.5 ± 0.5	23.3 ± 0.6	37 ± 2	35.8 ± 0.7	52 ± 6	58 ± 3
V662γ2	6.59 ± 0.07	6.94 ± 0.06	14.27 ± 0.08	13.9 ± 0.1	20.8 ± 0.1	19.89 ± 0.06	29.0 ± 0.3	28.1 ± 0.3
V664γ1	8.24 ± 0.06	8.53 ± 0.06	14.84 ± 0.09	14.4 ± 0.1	20.3 ± 0.1	19.42 ± 0.06	27.9 ± 0.2	27.3 ± 0.3
V664γ2	8.03 ± 0.08	8.24 ± 0.07	16.1 ± 0.1	15.3 ± 0.1	22.1 ± 0.2	20.62 ± 0.09	-	-
I674δ1	9.52 ± 0.06	10.57 ± 0.07	24.7 ± 0.2	25.6 ± 0.3	38.3 ± 0.9	38.0 ± 0.4	61 ± 5	56 ± 2
V677γ1	16.1 ± 0.1	16.8 ± 0.2	27.5 ± 0.4	30.3 ± 0.5	37.2 ± 0.8	41.5 ± 0.6	54 ± 2	59 ± 2
V677γ2	8.28 ± 0.08	8.84 ± 0.07	19.2 ± 0.1	19.9 ± 0.2	29.0 ± 0.4	29.4 ± 0.2	43 ± 1	45 ± 1
V682γ1	10.0 ± 0.1	7.22 ± 0.06	15.66 ± 0.08	14.8 ± 0.1	21.4 ± 0.1	21.05 ± 0.06	-	27.0 ± 0.2
V693γ2	10.1 ± 0.1	10.8 ± 0.1	30.4 ± 0.6	28.9 ± 0.6	42 ± 2	43 ± 1	67 ± 9	-
I695δ1	9.83 ± 0.07	10.39 ± 0.06	24.6 ± 0.2	24.6 ± 0.3	37.6 ± 0.7	37.2 ± 0.3	54 ± 1	52 ± 1
L701δ1	5.09 ± 0.09	5.22 ± 0.05	9.49 ± 0.07	9.5 ± 0.07	13.9 ± 0.1	13.19 ± 0.04	19.8 ± 0.2	18.9 ± 0.2
L701δ2	7.1 ± 0.2	8.6 ± 0.2	14.3 ± 0.3	15.2 ± 0.3	21.4 ± 0.5	21.2 ± 0.2	28 ± 1	29 ± 1
V702γ1	8.13 ± 0.06	7.93 ± 0.06	18.4 ± 0.1	17.8 ± 0.2	27.1 ± 0.3	24.9 ± 0.1	41.5 ± 0.7	30.8 ± 0.3
V702γ2	5.54 ± 0.07	6.16 ± 0.05	13.77 ± 0.09	13.28 ± 0.09	20.5 ± 0.1	20.47 ± 0.07	31.7 ± 0.5	33.1 ± 0.4
V703γ1	8.34 ± 0.08	8.53 ± 0.07	18.7 ± 0.1	17.9 ± 0.2	28.9 ± 0.4	26.6 ± 0.2	39 ± 1	41.1 ± 0.9
V703γ2	7.66 ± 0.09	8.2 ± 0.08	19.4 ± 0.2	20.4 ± 0.3	29.2 ± 0.6	31.5 ± 0.3	43 ± 2	56 ± 1
V707γ1	8.79 ± 0.07	9.56 ± 0.08	21.8 ± 0.2	22.8 ± 0.3	33.3 ± 0.5	32.6 ± 0.3	47 ± 2	50 ± 1
V707γ2	8.23 ± 0.08	8.99 ± 0.09	23.1 ± 0.3	23.3 ± 0.4	33.5 ± 0.8	35.8 ± 0.5	50 ± 3	52 ± 2
V717γ1	10.22 ± 0.08	11.4 ± 0.1	25.8 ± 0.4	27.2 ± 0.4	40 ± 1	40.8 ± 0.6	58 ± 4	59 ± 2
V717γ2	11.3 ± 0.1	12.9 ± 0.1	31.5 ± 0.6	30.4 ± 0.7	45 ± 2	46 ± 1	62 ± 8	71 ± 6
V718γ1	6.05 ± 0.06	5.94 ± 0.04	11.06 ± 0.05	10.91 ± 0.05	15.9 ± 0.09	14.93 ± 0.03	21.9 ± 0.1	20.4 ± 0.2
V718γ2	5.34 ± 0.06	5.13 ± 0.03	9.44 ± 0.04	9.41 ± 0.04	13.67 ± 0.08	12.96 ± 0.02	19.4 ± 0.2	18.1 ± 0.1
V723γ1	6.67 ± 0.06	6.15 ± 0.04	13.8 ± 0.07	13.13 ± 0.07	21.4 ± 0.1	20.33 ± 0.06	31.0 ± 0.3	29.6 ± 0.3
V723γ2	6.06 ± 0.06	5.49 ± 0.03	13.8 ± 0.08	13.35 ± 0.08	22.3 ± 0.2	21.24 ± 0.07	34.1 ± 0.5	32.6 ± 0.4
V729γ1	9.51 ± 0.09	10.5 ± 0.1	25.6 ± 0.4	26.1 ± 0.5	39 ± 1	38.6 ± 0.6	51 ± 3	45 ± 2
V729γ2	8.52 ± 0.09	9.4 ± 0.1	23.9 ± 0.3	25.7 ± 0.5	38 ± 1	38.7 ± 0.6	-	-
V731γ1	14.3 ± 0.1	15.9 ± 0.1	36.5 ± 0.9	38.9 ± 0.9	51 ± 3	46 ± 1	71 ± 7	-
L733δ1	11.0 ± 0.2	9.7 ± 0.1	23.1 ± 0.4	21.7 ± 0.4	32.6 ± 0.9	31.1 ± 0.4	45 ± 1	45 ± 2
L733δ2	7.77 ± 0.09	7.05 ± 0.08	14.0 ± 0.1	13.8 ± 0.1	19.8 ± 0.2	18.86 ± 0.07	27.4 ± 0.3	26.4 ± 0.4
I738δ1	8.63 ± 0.05	9.3 ± 0.06	21.9 ± 0.1	21.8 ± 0.2	33.9 ± 0.4	32.6 ± 0.2	49 ± 1	49 ± 1
I743δ1	4.6 ± 0.05	4.73 ± 0.03	9.38 ± 0.05	9.4 ± 0.04	13.45 ± 0.09	13.31 ± 0.03	19.4 ± 0.2	18.6 ± 0.1
V745γ1	8.0 ± 0.06	8.46 ± 0.06	16.8 ± 0.1	16.2 ± 0.1	24.1 ± 0.3	22.65 ± 0.09	32.4 ± 0.4	32.2 ± 0.5
I748δ1	4.36 ± 0.05	4.43 ± 0.03	8.85 ± 0.03	8.94 ± 0.04	13.55 ± 0.07	12.9 ± 0.02	20.3 ± 0.1	19.0 ± 0.1

Supplementary Table 2: Methyl ^{1}H R_2 for isolated FLN5 at glycerol concentrations of 0, 40, 50 and 60 % (w/w), acquired at 900 and 950 MHz, 298 K.

Methyl	0%	40%	50%	60%
L661δ1	8.2 ± 0.2	30.9 ± 0.4	48 ± 1	73 ± 2
L661δ2	5.5 ± 0.1	20.0 ± 0.3	30.8 ± 0.6	47 ± 2
V662γ2	5.73 ± 0.09	20.0 ± 0.2	28.9 ± 0.3	45.8 ± 0.5
V664γ1	2.84 ± 0.06	8.2 ± 0.1	11.5 ± 0.1	17.5 ± 0.2
V664γ2	2.8 ± 0.06	7.52 ± 0.07	11.2 ± 0.2	19.2 ± 0.4
I674δ1	6.3 ± 0.1	22.1 ± 0.3	33.1 ± 0.9	49 ± 1
V677γ1	6.6 ± 0.2	26.6 ± 0.4	42.0 ± 0.8	64 ± 1
V677γ2	7.5 ± 0.2	28.5 ± 0.3	44.1 ± 0.6	66.3 ± 0.9
V682γ1	5.19 ± 0.08	20.5 ± 0.3	32.2 ± 0.4	49.0 ± 0.8
V693γ2	6.2 ± 0.1	19.5 ± 0.4	26.4 ± 0.8	42 ± 2
I695δ1	5.5 ± 0.08	18.1 ± 0.2	26.8 ± 0.3	39.8 ± 0.7
L701δ1	2.72 ± 0.07	8.34 ± 0.07	11.2 ± 0.1	16.9 ± 0.2
L701δ2	3.1 ± 0.1	11.7 ± 0.2	17.3 ± 0.4	23.2 ± 0.8
V702γ1	6.3 ± 0.1	24.2 ± 0.2	38.0 ± 0.4	56 ± 1
V702γ2	4.51 ± 0.08	16.9 ± 0.2	24.8 ± 0.3	41.3 ± 0.4
V703γ1	4.8 ± 0.1	16.6 ± 0.2	23.6 ± 0.4	36.0 ± 0.7
V703γ2	6.8 ± 0.1	24.0 ± 0.3	36.7 ± 0.7	58.0 ± 0.9
V707γ1	7.2 ± 0.2	26.6 ± 0.3	40.7 ± 0.4	59 ± 1
V707γ2	6.7 ± 0.2	23.3 ± 0.3	34.8 ± 0.6	47 ± 1
V717γ1	7.1 ± 0.1	25.5 ± 0.3	38.9 ± 0.8	61 ± 1
V717γ2	8.7 ± 0.2	31.9 ± 0.3	46 ± 1	64 ± 4
V718γ1	3.54 ± 0.07	10.6 ± 0.1	14.6 ± 0.2	21.4 ± 0.2
V718γ2	2.85 ± 0.04	9.12 ± 0.05	11.9 ± 0.1	18.0 ± 0.2
V723γ1	1.36 ± 0.03	3.16 ± 0.08	5.8 ± 0.3	7.8 ± 0.1
V723γ2	1.72 ± 0.03	4.47 ± 0.09	6.4 ± 0.2	9.1 ± 0.2
V729γ1	7.7 ± 0.2	28.0 ± 0.3	42 ± 1	63 ± 3
V729γ2	8.0 ± 0.1	29.1 ± 0.4	43.4 ± 0.6	63 ± 2
V731γ1	8.1 ± 0.2	27.0 ± 0.5	39 ± 2	67 ± 5
L733δ1	4.2 ± 0.1	13.2 ± 0.2	21.5 ± 0.4	33.4 ± 0.7
L733δ2	4.2 ± 0.1	14.7 ± 0.2	21.5 ± 0.3	32.5 ± 0.5
I738δ1	6.7 ± 0.07	23.6 ± 0.2	36.2 ± 0.3	56 ± 1
I743δ1	3.17 ± 0.07	9.31 ± 0.04	13.9 ± 0.1	21.6 ± 0.2
V745γ1	3.43 ± 0.08	10.3 ± 0.1	14.7 ± 0.2	20.9 ± 0.4
I748δ1	3.77 ± 0.09	11.85 ± 0.06	17.3 ± 0.1	25.4 ± 0.2

Supplementary Table 3: Methyl $S^2_{axis}\tau_c$ values for isolated FLN5 at glycerol concentrations of 0, 40, 50 and 60 % (w/w) recorded at 900 and 950 MHz, 298 K.

Methyl	^1H R_2 (s^{-1})							
	+67 950 MHz	+67 GS 950 MHz	+67 E6 950 MHz	+67 K2 950 MHz	+47 900 MHz	+57 900 MHz	+67 900 MHz	+110 900 MHz
L661δ1	37.7 ± 4.7	35.0 ± 11.0	35.0 ± 8.1	-	-	70.2 ± 9.9	50.0 ± 7.2	37.3 ± 6.1
L661δ2	42.3 ± 5.5	42.0 ± 11.0	49.0 ± 20.0	-	-	88.0 ± 18.0	41.7 ± 6.8	41.5 ± 7.1
V664γ1	26.4 ± 0.61	28.5 ± 2.3	28.0 ± 1.4	46.8 ± 6.5	46.6 ± 7.7	42.7 ± 2.3	27.2 ± 1.2	20.58 ± 0.61
V664γ2	21.54 ± 0.65	-	-	-	-	-	-	20.86 ± 0.69
I674δ1	48.4 ± 3.3	45.5 ± 8.9	40.1 ± 5.3	-	-	57.3 ± 6.2	51.9 ± 4.6	35.5 ± 2.1
V677γ1	38.9 ± 2.5	43.4 ± 8.9	38.7 ± 2.8	-	-	58.0 ± 5.3	41.0 ± 5.9	36.6 ± 3.8
V677γ2	51.6 ± 6.7	-	42.0 ± 13.0	-	-	-	-	42.7 ± 6.4
I695δ1	44.3 ± 2.5	55.7 ± 8.6	49.3 ± 6.2	146.0 ± 30.0	-	70.1 ± 6.1	45.7 ± 3.4	33.7 ± 1.8
L701δ1	22.26 ± 0.76	21.1 ± 1.8	24.1 ± 2.9	82.0 ± 13.0	-	34.4 ± 2.3	20.8 ± 1.4	15.93 ± 0.62
L701δ2	39.3 ± 4.1	43.0 ± 17.0	-	-	-	57.7 ± 9.9	37.1 ± 6.5	27.5 ± 2.2
V702γ1	31.0 ± 1.0	-	27.2 ± 1.2	-	39.8 ± 2.5	-	-	-
V702γ2	28.98 ± 0.74	29.3 ± 2.3	18.6 ± 1.5	84.0 ± 10.0	-	50.7 ± 1.7	37.73 ± 0.91	21.43 ± 0.83
V703γ1	36.7 ± 2.1	31.9 ± 5.3	31.3 ± 2.4	83.0 ± 20.0	-	52.6 ± 5.8	37.6 ± 3.4	29.1 ± 2.3
V703γ2	50.2 ± 5.7	49.0 ± 15.0	33.9 ± 3.2	-	-	70.8 ± 6.2	43.8 ± 8.7	31.1 ± 2.8
V707γ1	27.5 ± 1.5	27.4 ± 4.1	-	-	-	-	-	-
V707γ2	48.0 ± 5.2	35.4 ± 9.3	34.4 ± 3.7	-	-	-	-	-
V717γ1	45.9 ± 7.4	27.0 ± 18.0	-	-	-	-	-	42.1 ± 5.0
V717γ2	56.0 ± 22.0	-	-	-	-	-	-	65.0 ± 21.0
V718γ2	20.52 ± 0.32	-	18.9 ± 1.7	-	-	-	20.84 ± 0.83	15.13 ± 0.47
V723γ1	16.76 ± 0.26	19.43 ± 0.62	15.45 ± 0.73	44.6 ± 2.3	34.6 ± 2.4	30.03 ± 0.71	17.42 ± 0.55	13.92 ± 0.15
V723γ2	18.52 ± 0.33	20.77 ± 0.96	20.3 ± 1.0	37.8 ± 3.3	36.8 ± 3.8	32.6 ± 1.2	18.94 ± 0.67	14.27 ± 0.37
V729γ1	49.5 ± 6.4	-	52.0 ± 10.0	-	-	77.0 ± 12.0	44.2 ± 10.0	32.4 ± 4.2
V729γ2	51.0 ± 12.0	-	-	-	-	-	-	49.0 ± 10.0
V731γ1	61.0 ± 16.0	-	-	-	-	-	-	41.7 ± 6.9
L733δ1	31.9 ± 1.4	35.7 ± 7.3	30.7 ± 2.6	85.0 ± 14.0	39.0 ± 23.0	65.1 ± 4.5	32.9 ± 2.6	24.6 ± 1.7
L733δ2	28.4 ± 1.1	36.3 ± 8.9	24.4 ± 2.8	64.0 ± 29.0	-	50.7 ± 5.4	32.4 ± 2.3	21.8 ± 1.0
I738δ1	38.1 ± 1.5	46.2 ± 6.3	38.0 ± 2.4	-	-	63.7 ± 3.5	41.3 ± 2.1	35.0 ± 1.6
I743δ1	25.29 ± 0.42	32.0 ± 2.0	22.2 ± 1.0	63.9 ± 3.9	50.0 ± 14.0	44.1 ± 1.5	25.07 ± 0.2	16.96 ± 0.33
V745γ1	31.5 ± 1.2	29.9 ± 4.2	31.8 ± 2.0	47.3 ± 9.8	-	45.5 ± 4.1	29.7 ± 1.8	22.6 ± 0.91
I748δ1	20.32 ± 0.31	21.06 ± 0.79	22.9 ± 1.1	81.0 ± 18.0	33.1 ± 4.2	37.1 ± 1.4	21.06 ± 0.64	14.77 ± 0.33

Supplementary Table 4: Methyl ^1H R_2 measurements for RNCs.

Methyl	$S^2_{axis}\ \tau_c$ (ns)							
	+67 950 MHz	+67 GS 950 MHz	+67 E6 950 MHz	+67 K2 950 MHz	+47 900 MHz	+57 900 MHz	+67 900 MHz	+110 900 MHz
L661δ1	33.7 ± 4.8	31.0 ± 11.0	31.0 ± 8.1	-	-	66.0 ± 11.0	45.7 ± 7.6	32.9 ± 6.3
L661δ2	63.7 ± 9.0	63.0 ± 18.0	74.0 ± 33.0	-	-	147.0 ± 33.0	65.0 ± 12.0	64.0 ± 13.0
V664γ1	13.63 ± 0.38	14.9 ± 1.4	14.59 ± 0.82	25.7 ± 3.8	28.7 ± 5.2	26.0 ± 1.6	15.62 ± 0.84	11.12 ± 0.41
V664γ2	12.8 ± 0.56	-	-	-	-	-	-	12.85 ± 0.66
I674δ1	45.2 ± 3.7	42.3 ± 9.0	36.9 ± 5.5	-	-	53.1 ± 6.4	47.7 ± 4.8	31.3 ± 2.2
V677γ1	63.7 ± 5.1	72.0 ± 17.0	63.3 ± 5.6	-	-	95.0 ± 10.0	65.0 ± 11.0	57.2 ± 7.1
V677γ2	66.0 ± 11.0	-	51.0 ± 21.0	-	-	-	-	43.6 ± 9.2
I695δ1	33.5 ± 2.3	42.7 ± 7.1	37.6 ± 5.2	116.0 ± 25.0	-	54.7 ± 5.3	34.7 ± 3.0	24.7 ± 1.6
L701δ1	20.4 ± 1.9	19.2 ± 2.4	22.3 ± 3.5	80.0 ± 16.0	-	34.5 ± 3.6	19.9 ± 2.0	14.6 ± 1.0
L701δ2	36.9 ± 5.0	40.0 ± 18.0	-	-	-	59.0 ± 12.0	35.8 ± 7.7	24.9 ± 2.9
V702γ1	40.7 ± 1.9	-	35.3 ± 2.0	-	54.2 ± 5.3	-	-	-
V702γ2	41.3 ± 1.8	41.8 ± 4.0	23.9 ± 2.6	133.0 ± 18.0	-	87.6 ± 5.0	62.9 ± 3.1	31.9 ± 1.9
V703γ1	48.6 ± 3.1	41.7 ± 7.7	40.8 ± 3.4	115.0 ± 28.0	-	62.0 ± 8.6	43.4 ± 5.2	32.9 ± 3.6
V703γ2	48.4 ± 6.8	47.0 ± 16.0	31.5 ± 3.8	-	-	73.0 ± 8.8	43.0 ± 10.0	29.7 ± 3.6
V707γ1	33.9 ± 2.2	33.8 ± 5.9	-	-	-	-	-	-
V707γ2	50.5 ± 5.9	37.0 ± 10.0	35.6 ± 4.1	-	-	-	-	-
V717γ1	48.1 ± 8.6	26.0 ± 21.0	-	-	-	-	-	41.7 ± 5.6
V717γ2	60.0 ± 25.0	-	-	-	-	-	-	72.0 ± 26.0
V718γ2	20.4 ± 1.1	-	18.6 ± 2.2	-	-	-	22.3 ± 1.3	15.16 ± 0.74
V723γ1	4.87 ± 0.29	5.64 ± 0.39	4.49 ± 0.33	12.9 ± 1.2	10.37 ± 0.95	9.02 ± 0.58	5.29 ± 0.31	4.25 ± 0.2
V723γ2	4.5 ± 0.13	5.1 ± 0.28	4.99 ± 0.3	9.64 ± 0.91	9.8 ± 1.1	8.64 ± 0.41	4.87 ± 0.22	3.58 ± 0.13
V729γ1	57.3 ± 8.1	-	61.0 ± 13.0	-	-	94.0 ± 16.0	52.0 ± 13.0	36.2 ± 5.6
V729γ2	64.0 ± 16.0	-	-	-	-	-	-	58.0 ± 13.0
V731γ1	48.0 ± 14.0	-	-	-	-	-	-	30.7 ± 6.4
L733δ1	40.1 ± 2.7	46.0 ± 11.0	38.3 ± 4.2	118.0 ± 22.0	51.0 ± 34.0	90.2 ± 6.9	42.5 ± 3.9	30.3 ± 2.6
L733δ2	18.0 ± 1.0	24.3 ± 7.2	14.8 ± 2.3	46.0 ± 23.0	-	36.8 ± 4.4	22.3 ± 1.9	13.76 ± 0.83
I738δ1	42.7 ± 2.1	52.6 ± 7.8	42.6 ± 3.1	-	-	76.7 ± 4.8	47.9 ± 2.9	39.8 ± 2.2
I743δ1	28.84 ± 0.77	37.1 ± 2.6	25.1 ± 1.3	76.5 ± 5.1	62.0 ± 18.0	53.7 ± 2.3	29.28 ± 0.7	18.88 ± 0.54
V745γ1	20.8 ± 1.1	19.7 ± 3.2	21.1 ± 1.6	32.6 ± 7.3	-	33.3 ± 3.6	20.6 ± 1.7	14.89 ± 0.91
I748δ1	27.2 ± 1.9	28.2 ± 2.2	30.9 ± 2.7	112.0 ± 27.0	48.4 ± 6.9	54.5 ± 3.5	29.9 ± 1.6	20.19 ± 0.85

Supplementary Table 5: Calculated methyl $S^2_{axis}\tau_c$ values for RNCs.

References

1. Hsu, S.-T. D., Cabrita, L. D., Fucini, P., Christodoulou, J. & Dobson, C. M. Probing side-chain dynamics of a ribosome-bound nascent chain using methyl NMR spectroscopy. *J. Am. Chem. Soc.* **131**, 8366–8367 (2009).
